# The impact of peer pressure on short video addiction of Chinese adolescents: a moderated mediation model

**DOI:** 10.1186/s40359-026-04529-y

**Published:** 2026-04-11

**Authors:** Xiaoping Deng, Wenyi Pan, Qing Lu, Yuzhen Wu

**Affiliations:** 1 School of Education Science, Gannan Normal University, Ganzhou, China; 2 Logistics Management Office, Gannan Normal University, Ganzhou, China

**Keywords:** Peer pressure, Short video addiction, Cognitive emotion regulation strategies, Social support

## Abstract

**Background:**

The rising popularity of short videos has sparked increasing worry about their potential for adolescent addiction. Guided by the ACE (Anonymity, Convenience, and Escape) and cognitive-behavioral model, our investigation examines how peer pressure is linked to this addiction, with specific attention to the mediating function of cognitive emotion regulation strategies and the buffering effect of social support.

**Methods:**

699 secondary school students completed an anonymous cross-sectional survey. Participants engaged with the Peer Pressure Questionnaire, Cognitive Emotion Regulation Questionnaire-Short, Adolescent Social Support Scale, and Short-Form Video Addiction Scale. We tested a moderated mediation model to analyze the data.

**Results:**

Results demonstrated that peer pressure was significantly associated with addiction. Maladaptive cognitive emotion regulation strategies partially mediated the association between peer pressure to short video addiction (β = 0.05, 95% CI [0.02, 0.08]). However, adaptive cognitive emotion regulation strategies did not demonstrate a significant mediating effect. Furthermore, social support was associated with a weaker impact of peer pressure on maladaptive strategies (β = -0.10, 95% CI [།0.17, །0.02]).

**Conclusions:**

These findings indicate that peer pressure is significantly associated with short video addiction through emotion regulation mechanisms, with social support exerting context-dependent associations. The research offers a theoretical basis for creating focused interventions to decrease short video addiction in adolescents.

## Introduction

The rising popularity of short videos has sparked increasing worry about their potential for adolescent addiction. Among adolescent internet users, 54.1% frequently watch short videos, with 11.9% spending more than two hours daily on this activity during weekdays, furthermore, 77.5% of parents report concern about their children’s excessive usage time [1]. Short video addiction is characterized by a persistent and profound psychological craving accompanied by compulsive use and dependent behavior directed towards short video applications [2]. This behavior is associated with both physical and mental health decline, posing risks for impaired vision, reduced fitness [3], difficulties in emotion regulation, reduced well-being [4], decreased academic engagement [5], and increased self-harm and suicidality [6, 7]. Consequently, understanding the psychological mechanisms and risk pathways to short video addiction holds considerable theoretical and practical importance.

Short video addiction, a specific subtype of social media addiction [8], shares the core diagnostic features of general internet addiction (e.g., withdrawal, salience, conflict, and relapse) common to disorders like gaming disorder [9]. A key differentiator is the shift from intentional, instrumental use to habitual, ritualistic use. This transition is largely driven by personalized recommendation algorithms, which continuously activate the brain’s reward system and trap users in an “algorithmic loop,” thereby reinforcing dependency [10]. Therefore, investigating short video addiction as a distinct phenomenon is crucial for accurately uncovering its unique psychological mechanisms and risk pathways.

Stress is a prominent predisposing factor in behavioral addictions [11, 12]. It is widely accepted that stress can be categorized based on its origin, with classification typically distinguishing between internal and external stressors. Ecological systems theory offers a theoretical model for comprehending how development is shaped by various environmental tiers, ranging from the direct microsystem to the wider macrosystem [13]. Of these, the microsystem—constituted by an proximal settings like family, school, and peer groups—plays a central role and is considered to have the most direct and profound impact on adolescents’ behavioral development. While previous studies have primarily examined family- and school-related stressors [14, 15], the association between peer pressure and short video addiction remains underexplored.

Peer pressure is one of the most salient social influences during adolescence. According to socialization theory [16, 17], as individuals progress through their life cycle, parental influence diminishes during the adolescent period, while peer influence grows stronger, especially under stressful conditions. Peer pressure is defined as the psychological stress experienced when an individual is influenced by peers to engage in behaviors inconsistent with their own intentions [18]. Negative peer pressure involves peers soliciting engagement in undesirable behaviors, whereas positive peer pressure entails encouraging positive behaviors or discouraging detrimental ones [19]. Given that this study uses short video addiction as the dependent variable and primarily focuses on its potential risk factors, the emphasis will be placed on examining peer pressure within the negative dimension. Within the ACE (Anonymity, Convenience, and Escape) model of internet addiction [20], online environments provide anonymity, convenience, and escapism, which can prompt adolescents to utilize short videos for coping with real-life stress. As a subtype of internet use [21], short video platforms are characterized by high engagement and instant gratification. This phenomenon has the potential to function as a psychological refuge for adolescents grappling with social interaction pressures. In addition, peer influence is associated with the prediction of internet addiction among adolescents [22]. Specifically, peer pressure may be linked to an increased risk of behaviors such as Internet addiction by exacerbating anxiety and depression [23].

To systematically elucidate the relationship between peer pressure and short video addiction among adolescents, it is essential to examine the underlying mechanisms. The present study proposes cognitive emotion regulation strategies, particularly maladaptive strategies, as a potential mediator. Cognitive emotion regulation strategies refer to the cognitive processes individuals use to manage emotionally charged information arising from stressful experiences [24]. These strategies are commonly classified into adaptive strategies (e.g., acceptance, positive refocusing, reengagement in planning, rational analysis) and maladaptive strategies (e.g., self-reproach, rumination, blaming others, catastrophizing) [25]. Maladaptive strategies, in particular, tend to intensify rather than alleviate negative emotional experiences [26, 27].

From a theoretical perspective, peer pressure represents a prevalent stressor during adolescence and is closely linked to emotional difficulties such as anxiety and depression [21, 28]. According to the process model of emotion regulation [26, 27], cognitive emotion regulation strategies operate primarily at the “cognitive change” stage, where individuals alter their interpretation of an emotional event to influence their emotional experience. Individuals under prolonged stress are more likely to adopt maladaptive strategies, which represent a failed cognitive change that amplifies and prolongs negative emotions [29, 30]. To cope with this intensified distress, individuals may shift to the “response modulation” stage, resorting to behavioral strategies to directly regulate their emotional responses. Short video platforms, characterized by high accessibility and immersive experiences [31], offer a readily available outlet for emotional escape; however, overreliance is significantly associated with addiction risk [32, 33]. Therefore, under peer pressure, adolescents may use maladaptive strategies as a cognitive pathway, increasing their likelihood of turning to short video use as a behavioral emotion regulation strategy, thereby heightening addiction risk.

From an empirical perspective, substantial research supports the association between stress and maladaptive cognitive emotion regulation. Chronic stress exposure increases the use of maladaptive strategies [29, 30], and adolescents experiencing peer pressure are particularly likely to employ strategies such as self-reproach and catastrophizing. More importantly, studies have confirmed that maladaptive cognitive emotion regulation strategies serve as a key mediating mechanism between stress and various behavioral addictions, including internet addiction [34, 35]. These findings provide empirical support for understanding how peer pressure may influence short video addiction via maladaptive cognitive pathways.

Integrating the theoretical and empirical perspectives above, it is plausible that peer pressure influences short video addiction among adolescents through the mediating role of maladaptive cognitive emotion regulation strategies. The cognitive-behavioral model of pathological internet use [36] provides a comprehensive framework for explaining this pathway, positing that both distal factors (e.g., peer pressure) and proximal factors (e.g., maladaptive cognition) jointly contribute to the development of addiction. Based on this, the present study constructs a moderated mediation model to examine the mediating role of maladaptive cognitive emotion regulation strategies in the relationship between peer pressure and short video addiction, as well as the moderating effect of social support within this pathway.

Social support may also alleviate such risks. According to stress-buffering theory, social support—defined as emotional, informational, or tangible resources from one’s social network—reduces the psychological impact of stress and promotes emotional stability [37]. In our model, this protection is hypothesized to occur via two pathways. First, social support is proposed to exert its protective role by moderating the relationship between peer pressure and cognitive emotion regulation strategies. Specifically, social support may help adolescents adopt adaptive cognitive emotion regulation strategies when facing peer pressure, while attenuating the positive association between peer pressure and maladaptive cognitive emotion regulation strategies [38]. Second, such support is hypothesized to have a direct moderating effect: by buffering the impact of stress, it may weakens the link between peer pressure and compensatory addictive behaviors. Specifically, well-supported adolescents facing peer pressure experience less emotional distress and have alternative ways to satisfy their needs, diminishing their reliance on short videos for escape or relief. Empirical evidence aligns with this, showing that family support weakens the stress-Internet addiction link [39]. The literature thus consistently corroborates the protective role of social support against stress-induced issues [40].

Despite these insights, prior research has been oriented towards family and school stressors regarding adolescent short video addiction [14, 15, 37], whereas peer pressure—one of the most salient social stressors during adolescence—has received insufficient attention regarding its association with short video addiction. To better understand the underlying mechanisms, this study introduces cognitive emotion regulation strategies as a mediator to examine their role in the relationship between peer pressure and short video addiction, and further investigates the moderating effect of social support. By constructing a moderated mediation model, this study aims to systematically elucidate the pathways and boundary conditions through which peer pressure influences short video addiction in adolescents.

**Fig. 1:**
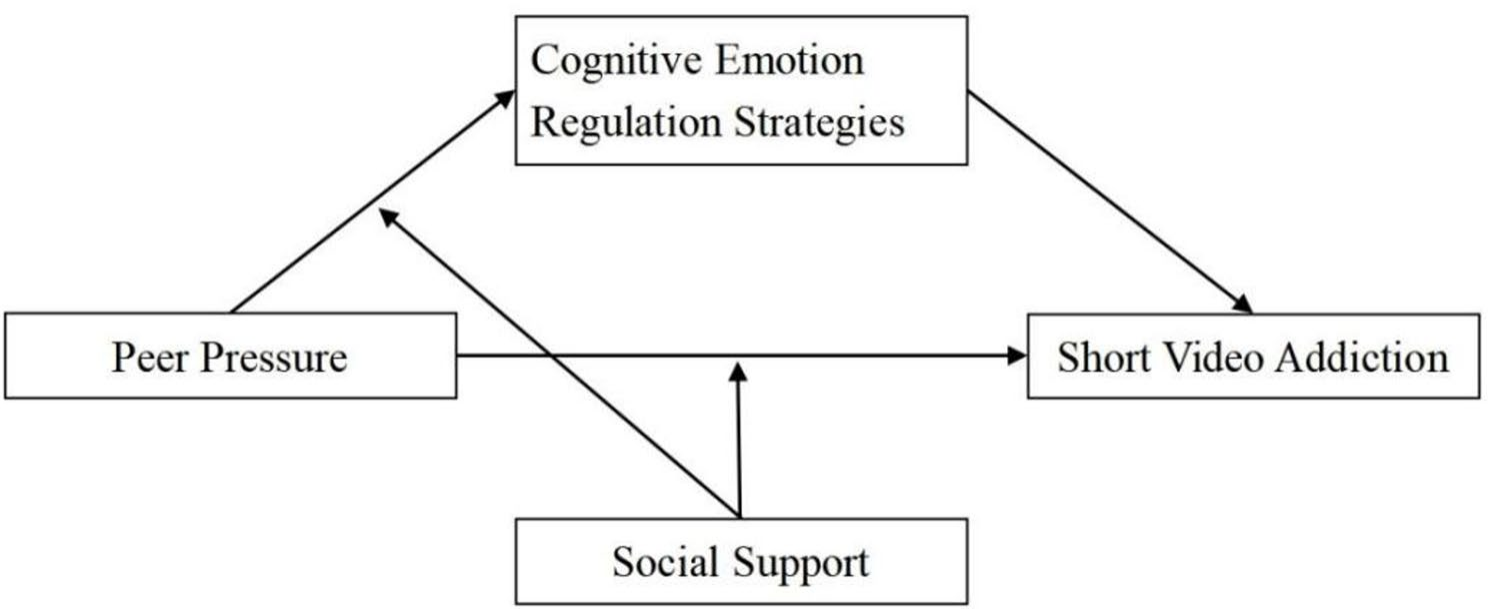
The research model

Informed by the extant literature and theoretical frameworks, we hypothesized the following (Fig. 1): H1, peer pressure positively predicts short video addiction. H2, CERS mediate this relationship. H3, Social support directly moderates the paths from peer pressure to CERS (H3a) and to short video addiction (H3b), such that high social support mitigates the relationships between peer pressure and CERS, in addition to the association between peer pressure and short video addiction.

## Method

### Participants

Data for this study were collected using an anonymous questionnaire survey. Participants were recruited from high schools in China. A total of 756 questionnaires were distributed. After excluding responses that failed the lie detection items, 699 valid questionnaires were retained, yielding an effective response rate of 92.46%. The participants’ ages ranged from 15 to 20 years (*M* ± *SD* = 16.25 ± 0.96). The sample consisted of 51.9% males (*N* = 347) and 48.1% females (*N* = 322). 400 students were in Grade 10, and 299 were in Grade 11. Due to their preparation for the college entrance examination, students in Grade 12 were excluded from the study. A 20-year-old 11th-grade student was enrolled due to re-enrollment after interruption. All participants took part voluntarily and gave informed consent prior to answering the questionnaires. Previous research has identified gender and age as significant factors influencing adolescent internet addiction; for instance, males show a significantly higher prevalence of internet addiction than females [41], and the main effects of both gender and age are significant [42]. Accordingly, gender and age were included as control variables in this study.

The data were collected between April 2025 and May 2025 through classroom-based group testing. Prior to data collection, written informed consent was obtained from the parents or legal guardians of all participating minors. In addition, informed assent was also obtained from the student participants themselves. Before data collection, the researchers elucidated the study’s objectives and procedures to all students, emphasizing that participation was anonymous, voluntary, and carried no financial or academic incentives. Participants were informed both in writing and verbally that they could decline without justifying and without negative consequences. The research protocol received ethical approval from the Ethics Review Committee of Gannan Normal University.

### Measures

The survey collected basic information related to participant gender, age, and grade.

Short-Form Video Addiction (SFVAS). Short video addiction was assessed using the 6-item Short-Form Video Addiction Scale [15]. This scale was adapted from the Social Network Service Addiction Scale [43] and has demonstrated good reliability and validity in Chinese adolescent samples [15, 44]. An example item is “Short video app usage distracts me from focusing on studying.” Responses were rated on a five-point Likert scale from completely inconsistent (1) to completely consistent (5). The mean score overall was calculated, with greater values suggesting a higher severity of short video addiction. The present study demonstrated remarkable internal consistency (α = 0.92).

Peer Pressure Questionnaire (PPI). Peer pressure was measured using the 26-item PPI [18, 45]. For each item, participants first identified whether their peers more frequently encouraged the behavior described on the left (negative peer pressure; e.g., “My peers often urge me to smoke”) or the opposite behavior on the right (positive peer pressure; e.g., “My peers often urge me not to smoke”). Items were rated on a seven-point Likert-type scale (-3 to 3), with positive scores reflecting negative peer pressure, negative scores reflecting positive peer pressure, and zero indicating no pressure. As the present study focused specifically on negative peer pressure as a risk factor, the scoring was adapted. Following procedures established in prior research [19], all original negative scores (which would indicate positive peer pressure) were recoded to 0 (“no positive pressure”). Subsequently, the remaining (negative peer pressure) scores were used, resulting in a unidirectional 4-point scale (0 = no pressure, 3 = much pressure). Higher scores thus indicated stronger perceived peer pressure. The present study demonstrated remarkable internal consistency (α = 0.93).

Cognitive Emotion Regulation Questionnaire-Short (CERQ-Short). We employed the 18-item CERQ short form to assess Cognitive-Emotional Regulation [25, 46]. The measure categorized strategies as either adaptive and MCERS. An example item is, “I feel that I am responsible for what has happened.” Responses were given on a five-point Likert-type scale from Never (1) to Always (5). In the present study, the internal consistency was acceptable for both ACERS (α = 0.78) and MCERS (α = 0.80).

Adolescent Social Support Scale. Social support was measured with the 17-item Adolescent Social Support Scale [47], comprising three dimensions: subjective support, objective support, and utilization of support. An example item is “When I encounter difficulties, I often seek help from family or relatives.” Responses were rated on a five-point Likert-type scale from completely inconsistent (1) to completely consistent (5), with higher scores indicating better perceived social support. The present study demonstrated remarkable internal consistency (α = 0.93).

### Data analyses

Analyses were conducted with SPSS 26 and the PROCESS macro (Version 4.2). To examine the mediating roles of ACERS and MCERS cognitive emotion regulation strategies, we first conducted separate mediation analyses using PROCESS Model 4 for each strategy. For the strategy that demonstrated a significant mediation effect, we then proceeded to test the moderated mediation effect using PROCESS Model 8, with social support as the moderator. The efficacy of the moderated mediation model was assessed through the implementation of a bootstrapping approach utilizing 5,000 samples. Before analysis, all continuous variables were standardized. Multicollinearity was not a concern (all VIF < 10; tolerance > 0.1).

## Results

### Descriptive analysis

Table 1 summarizes the descriptive statistics and intercorrelations of the study variables. Age was negatively correlated with short video addiction, and gender was negatively correlated with ACERS. Accordingly, age and gender were included as covariates in the moderating mediation model. Regarding the primary variables in the hypothesized model, peer pressure was significantly positively correlated with MCERS and short video addiction, and MCERS was significantly positively correlated with short video addiction. However, contrary to our theoretical expectations, ACERS was not significantly correlated with either peer pressure or short video addiction. This pattern suggests that in the context of peer pressure, maladaptive cognitive emotion regulation strategies may play a more prominent role than adaptive strategies in explaining the development of short video addiction, a finding that is further examined in the subsequent mediation analyses.

**Table 1:** Descriptive statistics and pearson correlations of the study variables (*N* = 669)

Variable	1	2	3	4	5	6	7
1.Peer Pressure	—						
2.MCERS	0.27^**^	—					
3.ACERS	0.05	0.48^**^	—				
4.Short Video Addiction	0.21^**^	0.20^**^	-0.02	—			
5.Social Support	-0.11^**^	-0.06	0.23^**^	-0.09^*^	—		
6.Gender	-0.08^*^	-0.01	-0.11^**^	-0.01	0.02	—	
7.Age	0.14^**^	-0.07	-0.05	0.13^**^	-0.02	-0.11^**^	—
*M* ± *SD*	0.45 ± 0.45	2.79 ± 0.65	2.96 ± 0.54	2.54 ± 0.98	3.30 ± 0.72		

*MCERS* Maladaptive Cognitive Emotion Regulation Strategies; *ACERS* Adaptive Cognitive Emotion Regulation Strategies. ^*^p < 0.05, ^**^p < 0.01

### Mediating effect of CERS

**Fig. 2:**
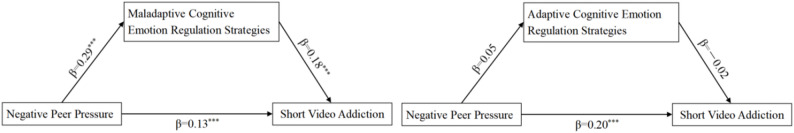
The mediating effect of cognitive emotion regulation strategies on the link between peer pressure and short video addiction. ( ^*^p < 0.05, ^***^p < 0.001)

Mediation analyses (PROCESS Model 4) were conducted to examine the mechanism through which peer pressure affects short video addiction through CERS.These analyses were performed separately for ACERS and MCERS, with all path coefficients and significance levels presented in Fig. 2.

As illustrated in Fig. 2, peer pressure was significantly associated with MCERS and short video addiction. MCERS, in turn, was significantly linked to short video addiction. These findings indicate that MCERS mediated the relationship between peer pressure and short video addiction (β = 0.05, 95% CI [0.02, 0.08]). However, the mediating path through ACERS was not significant. Specifically, the indirect effect of peer pressure on short video addiction via ACERS was not significant (β = -0.001, 95% CI [-0.01, 0.002]). Given the nonsignificant mediation effect of ACERS, the moderated mediation analysis was not pursued further for this pathway.

### Moderating effect of social support

To explore the moderating function of social support, we conducted a moderated mediation analysis using PROCESS Model 8 for the pathway involving MCERS, given its significant mediation effect.

**Fig. 3:**
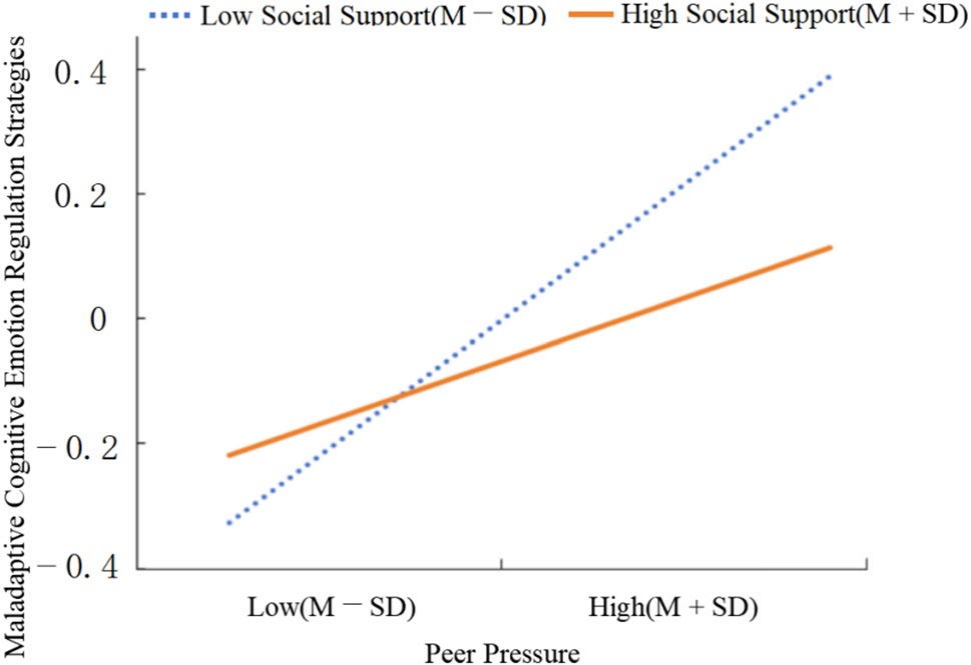
The conditional effects of social support on the relationship between peer pressure and Maladaptive cognitive emotion regulation strategies

In the model incorporating peer pressure, the interaction term (Peer Pressure × Social Support) significantly negatively predicted MCERS (β = -0.10, *p* = 0.016), but not short video addiction directly (Table 2). Simple slope analysis (Fig. 3) further revealed that the effect of peer pressure on MCERS varied across different levels of social support. Specifically, at low levels of social support (*M*-*SD*), peer pressure was strongly associated with MCERS (β = 0.31, SE = 0.06, *p* < 0.001, 95% CI [0.20, 0.42]). At high levels of social support (*M* + *SD*), the association between peer pressure and MCERS was weaker (β = 0.12, SE = 0.06, *p* = 0.04, 95% CI [0.01, 0.24]).

**Table 2:** Testing the moderated mediation in the peer pressure model

Predictors	On MCERS					On Short Video Addiction				
	β	SE	*p*	95% CI		β		SE	*p*	95% CI
Gender	-0.01	0.07	0.939	[-0.15, 0.14]		0.04		0.08	0.634	[0.11, 0.18]
Age	-0.11	0.04	0.004	[-0.19, །0.04]		0.13		0.04	0.001	[0.05, 0.20]
Peer Pressure	0.28	0.04	< 0.001	[0.20, 0.35]		0.15		0.04	< 0.001	[0.07, 0.22]
Social Support	-0.04	0.04	0.258	[-0.12, 0.03]		-0.06		0.04	0.088	[-0.14, 0.01]
MCERS						0.17		0.04	< 0.001	[0.09, 0.25]
Peer Pressure×Social Support	-0.10	0.04	0.016	[-0.17, །0.02]		0.05		0.04	0.218	[-0.03, 0.13]
R^2^	0.09					0.09				
*F*	13.58					10.65				

*MCERS* Maladaptive Cognitive Emotion Regulation Strategies. *N* = 669

## Discussion

The present study investigates the mechanisms linking peer pressure to short video addiction, focusing on CERS as the mediating mechanism and social support as a key moderating factor. These findings confirm that MCERS mediate the association between peer pressure and addiction and that social support moderates this pathway.

### Peer pressure and short video addiction

Hypothesis 1 was validated, with peer pressure being significantly associated with higher levels of short video addiction [48]. This phenomenon can be explained through the “escape” mechanism of the ACE model [20], whereby adolescents may turn to short videos to cope with anxiety and frustration linked to peer pressure, potentially seeking emotional relief [28].

### Mediating role of CERS

Supporting Hypothesis 2, MCERS partially mediated the relationship between peer pressure and short video addiction. Peer pressure was significantly associated with the use of MCERS, which in turn was positively correlated with short video addiction. This finding aligns with the transactional model of stress appraisal [49], which suggests that stressful environments or negative events do not directly lead to adverse psychological outcomes but operate through an individual’s cognitive evaluation of the stressors. Negative appraisals and irrational interpretations—reflected here as maladaptive cognitive emotion regulation—can amplify the detrimental effects of stress, contributing to emotional disturbances and behavioral issues such as addiction. Empirical studies further indicate that individuals under high levels of stress are more prone to develop maladaptive cognitions [50, 51], which may explain why peer pressure is more likely to facilitate short video addiction via MCERS rather than through adaptive strategies.

In contrast, ACERS did not demonstrate a significant mediating role in the link between peer pressure and addiction. This may be because ACERS focus more on internal emotional management and cognitive restructuring [52], which are oriented toward enhancing autonomous emotion regulation capacity and fostering flexible adjustment of cognitive and behavioral patterns. Their protective effects are thus more likely to manifest in improving overall emotional resilience rather than directly counteracting the pathway to addictive behavior [53]. In other words, while adaptive strategies help individuals cope with stress in a healthier manner, they do not necessarily intervene in the specific cognitive-behavioral route that links peer pressure to compulsive short video use.

### Moderating role of social support

This study revealed that social support mitigates the association between peer pressure and MCERS, a result that supports the stress-buffering theory and prior studies showing that family support is linked to lower stress-induced internet addiction [39]. This underscores its protective role at the cognitive appraisal stage against stress. This buffering effect can be understood through the mechanism of cognitive reappraisal. Supportive networks may help adolescents reframe peer pressure as a manageable challenge rather than an uncontrollable threat. This positive reappraisal reduces initial negative emotional arousal, thereby diminishing the reliance on immediate, avoidant coping strategies like rumination or catastrophizing (i.e., MCERS). This aligns with evidence suggesting that social support primarily operates by buffering the cognitive appraisal of stressful events and the subsequent emotional response [54], effectively intercepting the stress process at a relatively proximal stage.

However, the same interaction (peer pressure × social support) did not significantly moderate the direct path from peer pressure to short video addiction. This nuanced finding may be explained by two interrelated factors. First, the nature of the support source matters. Our measure of social support likely amalgamated different sources, such as family and peer support. Research suggests these sources may play divergent roles in the context of behavioral addiction. For instance, family support is consistently linked to buffering the impact of stress on addiction [55]. In contrast, friend support in adolescence, while emotionally valuable, might not buffer addictive behaviors [55]. In the context of negative peer pressure, support from friends who themselves engage in high levels of short video use might provide emotional comfort (explaining the buffer on MCERS) but simultaneously model and normalize the compensatory behavior (weakening a direct buffer on addiction). Second, the level of influence differs. MCERS represents an internal, cognitive-emotional process highly susceptible to the affective and appraisal-shifting qualities of general social support [54]. Conversely, the leap from stress or negative cognition to a sustained addictive behavior is governed by a more complex array of factors, including ingrained habits, immediate environmental cues, and the potent reward system of the platforms themselves. Therefore, social support’s primary protective role in this model appears to be preventing the generation of maladaptive cognitive patterns under stress, rather than directly disrupting the established link between stress and addictive behavior once those patterns are active.

### Practical implications

Building directly on these findings, targeted interventions should prioritize three components. First, reduce exposure to and build resilience against negative peer pressure through social-emotional learning in schools. Second, and most critically, directly target and train adolescents to reduce MCERS, such as rumination, via specific cognitive-behavioral modules rather than only promoting generic adaptive coping. Third, strategically strengthen family support, as it buffers the initial stress-appraisal stage, while carefully navigating the complex role of peer support to avoid reinforcing addictive habits. A multi-component approach addressing this social risk, cognitive mechanism, and key protective factor is recommended.

### Limitations

There are several limitations to the study. First, the causal interpretation is limited by the cross-sectional survey. Future research should adopt longitudinal or experience-sampling methods to explore dynamic interactions among these variables. Secondly, future studies ought to clearly distinguish between the diverse sources of support (e.g., family vs. peer) and further examine how these distinct sources interact dynamically with different types of distinct CERS.

## Conclusion

This study highlights the complex pathways through which peer pressure is linked to short video addiction among adolescents. Peer pressure was directly associated with short video addiction and indirectly associated via MCERS. Moreover, social support significantly attenuated the association between peer pressure and MCERS.

## Data Availability

The data that supported the findings of this study are openly available in Mendeley Data at https://data.mendeley.com/preview/bsdwt3r5ts? a=1652caa7-577b-4068-b0a2-e429fee0b46d.

## Abbreviations

CERS: Cognitive Emotion Regulation Strategies
ACERS: Adaptive Cognitive Emotion Regulation Strategies
MCERS: Maladaptive Cognitive Emotion Regulation Strategies
